# Green additives effectiveness in water-based drilling mud performance: A study of pistachio shell powder, magnesium oxide, and carbon black particles

**DOI:** 10.1371/journal.pone.0357783

**Published:** 2026-09-21

**Authors:** Vamsi Krishna Kudapa, Wilson Kosasih, A.R. Saravanan, Amaleswari Rajulapati, K. Kumar, Vijayakumar Sivasundar, Din Bandhu, Ankit Sharma, Ashish Kumar, Najihah Mohd Tamyis

**Affiliations:** 1 Department of Chemical Engineering, School of Advanced Engineering, UPES, Dehradun, India; 2 Department of Industrial Engineering, Universitas Tarumanagara, Jakarta, Indonesia; 3 Department of Aeronautical Engineering, KIT-Kalaignarkarunanidhi Institute of Technology, Coimbatore, Tamil Nadu, India; 4 Department of Electrical and Electronics Engineering, Academy of Maritime Education and Training (AMET) Deemed University, East Coast Road, Kanathur, Chennai, India; 5 Department of Mechanical Engineering, Inderprastha Engineering College (IPEC), Ghaziabad, India; 6 Universiti Kuala Lumpur, Malaysian Institute of Aviation Technology (UniKL MIAT), Lot Jalan Jenderam Hulu, Dengkil, Selangor, Malaysia; 7 Department of Mechanical Engineering, Saveetha School of Engineering, SIMATS, Saveetha University, Chennai, Tamil Nadu, India; 8 Additive Manufacturing Research and Innovation Center (AMRIC), Mechanical Engineering Department, College of Engineering, Prince Mohammad Bin Fahd University, Al-Khobar, Saudi Arabia; 9 Centre for Research Impact & Outcome, Chitkara University Institute of Engineering and Technology, Chitkara University, Rajpura, Punjab, India; 10 Division of Research and Development, Lovely Professional University, Phagwara, India; University of Vigo, SPAIN

## Abstract

Drilling fluid performance is critical for wellbore stability, cuttings transport, and minimizing formation damage, but conventional additives often raise environmental concerns. This study evaluates the effectiveness of three additives, pistachio shell powder (PSP), magnesium oxide (MgO) nanoparticles, and carbon black (CB), in enhancing the rheological and filtration properties of water‑based drilling muds (WBMs). Ten mud formulations with varying additive concentrations (1–5 g) were prepared, and key parameters including density, pH, plastic viscosity, yield point, gel strength, API fluid loss, and shear‑dependent viscosity were systematically characterized. Results show that PSP significantly improved rheological performance, increasing yield point to 0.771 kg/m² and gel strength to 19.6 lb/100 ft², while maintaining stable density (9.82–9.88 ppg) and pH (8.0–8.2). In filtration tests, PSP achieved an API filtrate volume of 22 mL, superior to CB (26–27 mL) and comparable to MgO, owing to the formation of a thin, low‑permeability filter cake. All muds exhibited non‑Newtonian shear‑thinning behaviour, with PSP offering an optimal balance between viscosity for cuttings suspension and flowability during circulation. As an agricultural waste‑derived additive, PSP demonstrates technical performance on par with MgO nanoparticles while being cost‑effective and environmentally suitable. The main goal of this research is to comparatively study the effects of pistachio shell powder (PSP), magnesium oxide (MgO) nanoparticles, and carbon black (CB) on the rheological and filtration characteristics of water-based drilling muds under similar experimental conditions, and to identify a sustainable and economical drilling fluid additive.

## 1. Introduction

Drilling fluids, also known as drilling muds, are critical to petroleum drilling. They maintain a steady hydrostatic pressure, stabilise the wellbore walls, transport surface cuttings, cool and lubricate the drill bit, and minimise formation damage. They also limit fluid loss and increase well-bore stability by creating a thin, low-permeability filter cake [1,2]. Constant contact with the formation and drilling equipment requires strict control of rheological and filtration characteristics [3]. The most common ones are water-based muds (WBM), which are more affordable, easy to prepare, and less harmful to the ecosystem. They are easy to dispose of and treat, thereby allowing their use at sensitive sites. However, as the industry advances to higher levels of both geometry and complexity, maintaining WBM performance under harsh downhole conditions remains a significant area of research interest [4]. Despite their popularity, traditional water-based muds (WBMs) are limited in their operational range. Over-intrusion of the filtrate leads to flow loss, decreased permeability, damage to the forms, and instability. Unrestrained invasion of filtrate may cause selective sticking, increased torque and drag, longer drilling time, and increased expenditure. Another issue is maintaining a constant rheological behaviour under changing conditions. The transport of cuttings is hindered by low viscosity or a yield point below the cuttings transport threshold, resulting in ineffective hole cleaning and the risk of pipe sticking. An increase in viscosity raises pumping pressure and energy consumption, reducing efficiency [5,6].

Water-based drilling fluids (WBMs) tend to swell upon contact with reactive formations. The swelling leads to well-bore instability, expansion, and potential collapse. A major design issue is the control of filtration, rheological stability and inhibition of WBMs. These properties can be improved by the use of chemical additives, including polymers, viscosifiers, and fluid-loss control agents [7]. The new breed of additives, enabled by nanotechnology, is nanoparticles. High performance is provided by their high surface area and the forces they generate. Alumina, silica, titania, zinc oxide, and magnesium nanoparticles are promising because they enhance viscosity, yield point, gel strength, and high-temperature stability [8]. They generate a fine-particle network and line pores, enhancing filtration and reducing damage to the formation.

At the same time, advances in nanotechnology have led to a greater commitment to developing environmentally friendly drilling fluid additives based on renewable resources [9]. Biodegradable substitutes for traditional chemicals include agricultural wastes, plant materials, and other biomaterial-based products. Substances such as rice husk ash, walnut shell bark powder, plant extracts, and lignocellulosic biomass have been explored for their capacity to improve filter cake quality and reduce fluid loss, owing to their fibrous or porous structure [10]. Furthermore, bio-derived additives enhance the rheology of drilling fluids by boosting viscosity and yield strength through interactions with natural polymers. These trends can be attributed to the continued shift towards sustainable, environmentally friendly drilling technology [11].

New developments in drilling fluids engineering have focused on improving rheological stability and filtration control. These efforts use nanomaterials and greener additives [12]. Nanoparticles have a high surface area and strong interparticle interactions, which greatly enhance drilling fluid performance. Al_2_O_3_, MgO, TiO_2_, and CuO metal oxide nanoparticles have been shown to increase rheology and hole-cleaning in water-based muds. MgO, in particular, increases the yield point and drilling performance [13]. Nano-silica has been reported to enhance flow behaviour and electrical stability during high temperatures [14]. AMPS/AM nano-plugging effectively reduces fluid loss and closes micro-fractures in HPHT conditions [15].

Similar studies on additives derived from biomass and agricultural waste emphasise their biodegradability and affordability. Rice husk ash exhibits better rheological and filtration properties [16]. Tea leaf extract can improve lubricity and reduce filter cake thickness [17]. Bio-based systems with vegetable oils or biodiesel exhibit favourable rheological and filtration properties and improved biodegradability [18,19]. Mixed environmentally friendly fluids containing green-synthesised nanoparticles and biomass further enhance filtration and mudcake quality [20]. Pistachio shell powder (PSP) is a potential sustainable, environmentally friendly substitute for traditional drilling additives, as it is a renewable, agricultural waste material and less environmentally hazardous [21]. Despite these developments, few comparative studies exist on nanoparticle and bio-derived additives under uniform experimental conditions. Their performance remains uncertain in comparison. Specifically, pistachio shells are a potentially useful agricultural waste product for use in drilling fluids, but they are little studied. Pistachio shells are produced in large quantities as byproducts of the food industry and are often considered waste. Their porous morphology, fibrous structure, and natural polymer composition suggest potential as fluid-loss control agents in drilling fluids [22–24]. However, systematic research comparing PSP with better-studied nanoparticle additives, such as MgO and CB, is lacking. To address this gap, this research explores the behaviour of PSP, MgO, and CB particles as additives in water-based drilling mud systems. Its novelty lies in a comparative assessment of bio-derived agricultural wastes and conventional inorganic nanoparticles, each at equal amounts, in a drilling fluid formation. The experiment examines how varying additive concentrations affect drilling fluid characteristics, including density, pH, rheology, shear-rate-activated viscosity, and API filtration patterns. The main aim of this work is to compare and analyse the performance of additives such as PSP, MgO and CB under the same condition and to check the performance of the additives to improve the rheological stability, filtration control, environmental compatibility etc. of WBMs.

## 2. Materials and methods

### 2.1. Testing apparatus

Different sets of equipment and instruments were used to prepare the mud sample and additives such as PSP, and to estimate the required properties of the resultant water-based drilling formulations. The list of instruments and equipment used for this experiment includes a digital Brookfield viscometer, a Hamilton Beach mixer, a Thermometer, beakers, a stopwatch, a weighing balance, a nitrogen cylinder, an OFITE LP-LT (API) filter press, a pH meter, a ball mill, an Antonpar rheometer, and a mud balance.

### 2.2. Materials

The drilling mud is prepared at the University of Petroleum and Energy Studies, India (UPES), along with some additives such as barite, xanthan gum, sodium hydroxide (NaOH), sodium chloride (NaCl), PSP, MgO nanoparticles, and carbon black compressed particles.

### 2.3. Pistachio shell powder preparation

**Fig 1:**
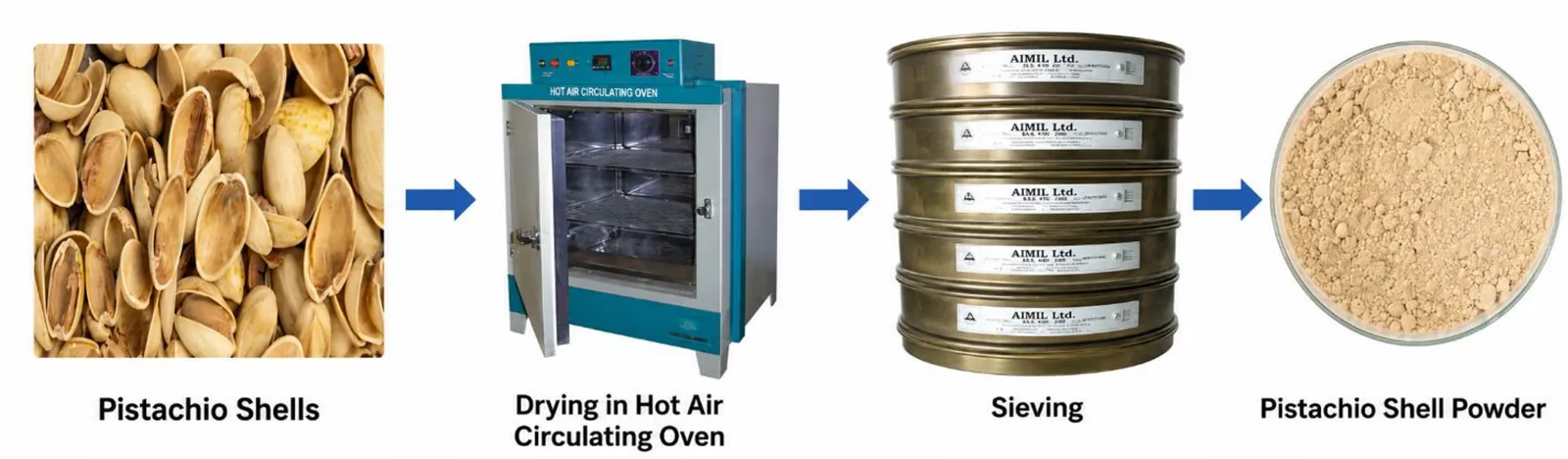
Schematic representation of fine pistachio shell powder preparation.

Pistachio nuts are well-known and widely available around the world; their shells are discarded as waste, and around 1 million tonnes/year of pistachio nuts were produced in 2019 [23]. For the present research work, pistachio nuts were collected from Dehradun, India, and the nut shells were initially washed, dried, and crushed, then placed in a hot air oven for 12 h at 105 °C to remove moisture from the crushed shells. The moisture removed from crushed Pistachio shells is milled using a ball mill and sieved to 250 nm (Fig 1).

**Fig 2:**
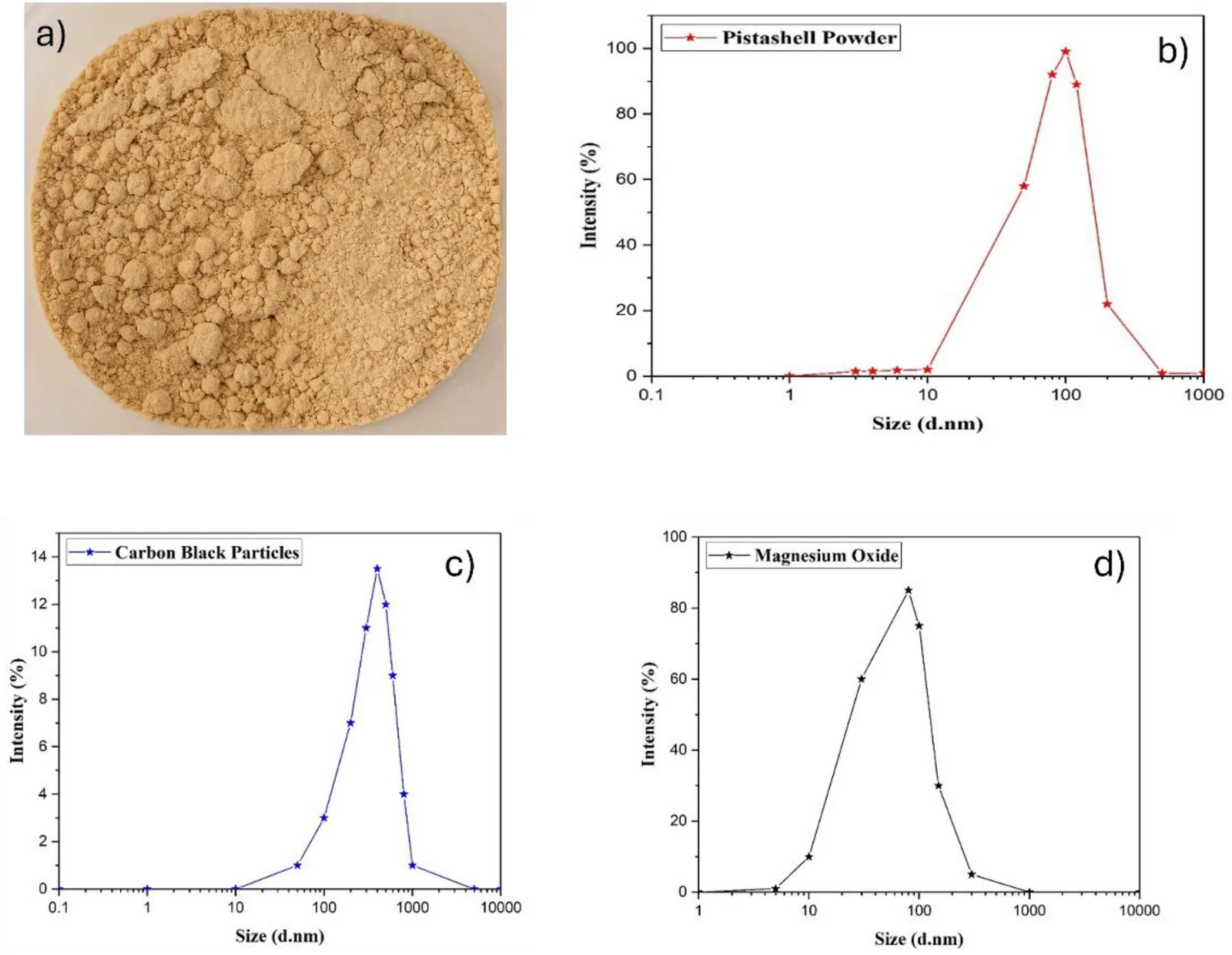
(a) Pictorial representation of prepared Pistachio shells powder; (b) depicts the pista shell particle size; (c -d) Particle size distribution profile of MgO and CB nanoparticles determined using particle size analysis to evaluate dispersion suitability for drilling fluid applications.

The pictorial representation of the ground pistachio shell powder is shown in Fig 2(a). The particle size distribution of the sample was analysed using the ZEN1690 particle size analyser, which operates on the principles of dynamic light scattering (DLS). The measurements were conducted under controlled conditions to ensure accuracy and reproducibility. The sample was dispersed in a suitable solvent, sonicated to achieve a uniform suspension, and then measured. The size distribution curve was unimodal, indicating a pure, homogeneous dispersion. The obtained data indicate that the sample has the desirable particle-size characteristics for use as a drilling fluid (Fig 2(b)).

### 2.4. Magnesium oxide

Magnesium oxide (MgO) is procured from Ad-Nano Technologies with a stated purity of >99% and an average particle size of 100 nm. The MgO nanoparticles were high-purity and exhibited a specific surface area of 60–90 m^2^/g. The nanoparticle sample was used as received without further purification. Before experimental use, the powder was stored in a desiccator to prevent moisture absorption and agglomeration. Characterization of the particle size was conducted using a Malvern particle analyser to verify the manufacturer’s specifications (Fig 2(c)).

### 2.5. Carbon black

Carbon Black nanoparticles are procured from Alfa Aesar with a stated purity of >99% and an average particle size of 100 nm. The carbon black nanoparticles were high-purity and exhibited a specific surface area of 75 m^2^/g. The nanoparticle sample was used as received without further purification. Before experimental use, the powder was stored in a desiccator to prevent moisture absorption and agglomeration. Characterization of the particle size was conducted using a Malvern particle analyser to verify the manufacturer’s specifications (Fig 2(d)).

### 2.6. Mud sample preparation

**Fig 3:**
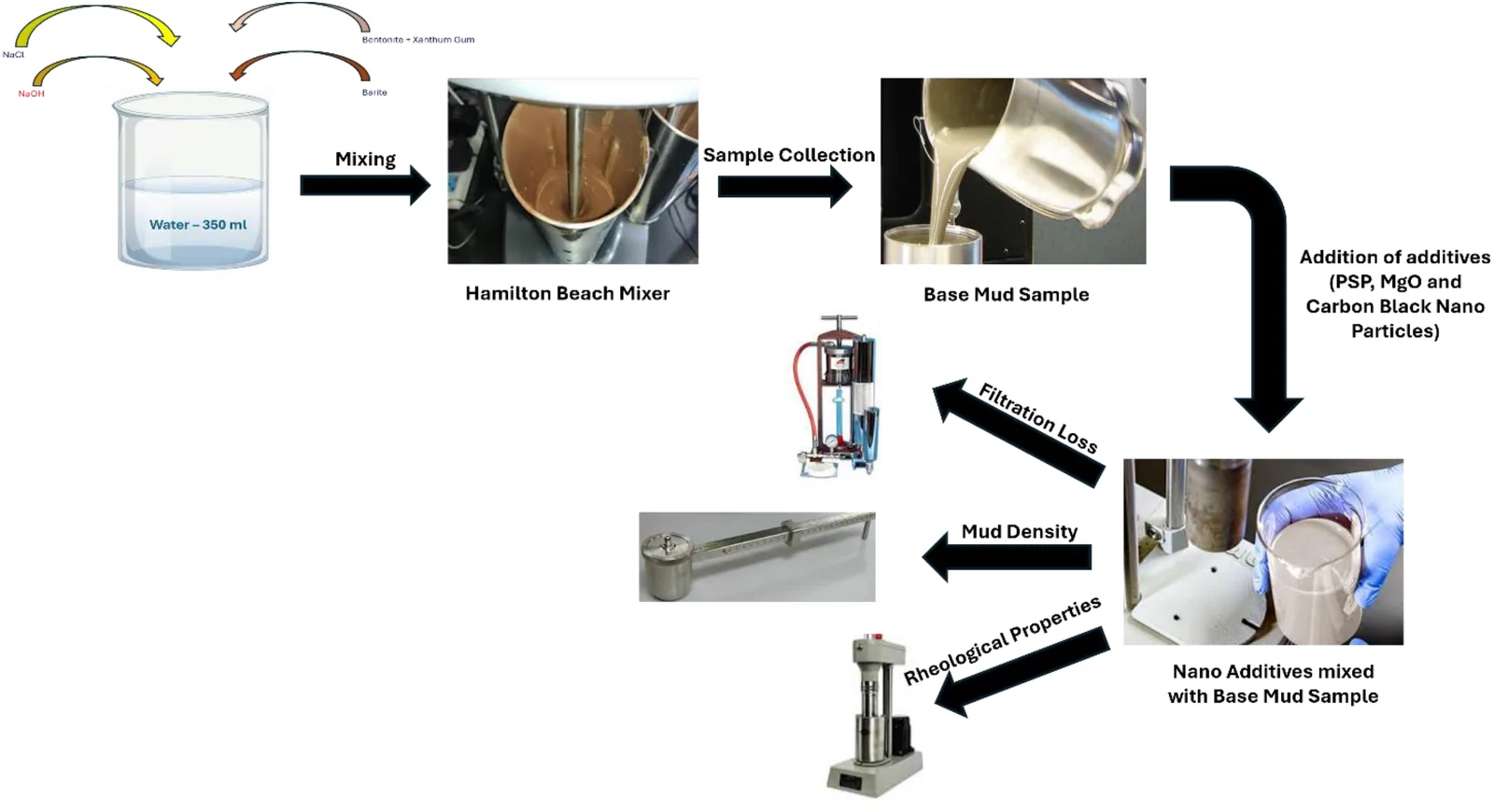
Schematic representation of nano-based drilling fluids and the properties estimated.

An optimised water-based mud was designed and formulated by adding various additives. The vessel was washed and cleaned, and 350 mL of water was added. Then, 5 g of caustic soda (NaOH) was added and mixed for 10 min to achieve the desired pH. Afterwards, sodium chloride (NaCl) was added and mixed for 10 min to control shale swelling. Then, for the next step, bentonite and xanthan gum were added and dispersed completely within 15 minutes to achieve a viscosity of 0.0285 Pa·s, thereby imparting water loss control. Subsequently, 10g of barite was added to the mud to achieve a higher-density mixture. A total of 10 drilling mud samples were formulated containing different concentrations of PSP (S1-S3), MgO nanoparticles (S4-S6), and CB (S7-S9). Variable concentrations of different nanoparticle materials were used to check the filter cake thickness, density and rheological behaviour (Fig 3). A mixing speed control was used on the Hamilton Beach mixer to prepare mud samples. The individual components were then added one at a time and mixed for 10−15 min until evenly distributed. To reduce agglomeration, nanoparticles were gradually added. All experiments were carried out at room temperature (25 ± 2 °C), and repeated three times to assure the reproducibility of the measurements. API filtration and rheological tests were conducted following the API Recommended Practice 13B-1, Water-based Drilling Fluids. The components and concentrations of the mud sample are listed in Table 1.

**Table 1:** Formulation of drilling fluid samples of ten different muds in SET A, SET B, and SET C.

Mud Component	Unit	Base Mud	SET A (PSP)			SET B (MgO)			SET C (CB)		
			S1	S2	S3	S4	S5	S6	S7	S8	S9
Water	mL	350	350	350	350	350	350	350	350	350	350
Bentonite	g	20	20	20	20	20	20	20	20	20	20
Barite	g	10	10	10	10	10	10	10	10	10	10
Xanthum Gum	g	1	1	1	1	1	1	1	1	1	1
NaOH	g	5	5	5	5	5	5	5	5	5	5
NaCl	g	5	5	5	5	5	5	5	5	5	5
PSP	g	–	1	2	5	–	–	–	–	–	–
MgO	g	–	–	–	–	1	2	5	–	–	–
CB	g	–	–	–	–	–	–	–	1	2	5

The concentration range chosen (1–5 g) was based on preliminary optimisation and literature data, as a drop in concentration level poses a risk of excessive viscosity, which could affect circulation efficiency and pumping requirements.

Table 1 features three SETs of drilling fluid samples, such as SET-A, SET-B, and SET-C. They include three S1 to S9 drilling fluid samples containing three different concentrations of PSP, MgO, and CB. PSP showed the best performance among the three additives in enhancing the characteristics of  water-based drilling muds under LTLP conditions, with the maximum reduction in filter cake thickness. On the other hand, MgO and CB also exhibited beneficial effects, and the optimal effective concentration varied with each evaluated property.

## 3. Results and Discussions

### 3.1. The effects of PSP, MgO, and CB Particle concentration on the density and pH of drilling mud are analysed

Drilling fluid parameters, such as density and pH, are also significant factors in controlling hydrostatic pressure and chemical stability during drilling. The experiment's findings demonstrate that adding PSP and CB has only a slight effect on total mud density and alkalinity. The density values did not vary by more than 0.06 ppg (9.82–9.88 ppg), and the pH was fairly constant at 8.0–8.2, indicating that the additives pose no threat to the chemical environment of the base mud. This could be explained by the relatively low specific gravity of PSP and CB, and by their low chemical activity with the base fluid's constituents. The same has been observed with biomass-based additives such as rice husk ash; the material primarily serves as a structural modifier and has no meaningful impact on the ionic makeup of the drilling fluid [16]. Table 2 shows the experimental values of density and pH.

**Table 2:** Density and pH values of base mud and drilling fluid sample formulation for ten different muds, SET A, SET B and SET C.

Mud Property	Unit	Base Mud	SET A (PSP)			SET B (MgO)			SET C (CB)		
			S1	S2	S3	S4	S5	S6	S7	S8	S9
Density	Ppg	9.83	9.82	9.83	9.83	9.88	10.23	10.88	9.84	9.82	9.84
pH	–	8.2	8.0	8.1	8.1	8.2	8.2	8.2	8.1	8.0	8.0

On the contrary, adding MgO nanoparticles led to a medium mud density, with the highest at 10.88 ppg at the highest concentrations. This is mainly due to the increased specific gravity and alkalinity of MgO nanoparticles. Its alkalinity also helps MgO enhance shut-in of shales and well-bore stability by maintaining a favourable chemical environment within the drilling fluid system. Similar behaviour has been reported for metal oxide nanoparticles in the past, with the comparatively high density of these nanoparticles and their high surface activity slightly increasing the density of drilling fluids but dramatically enhancing their stability and inhibition ability [13,24]. In general, the outcomes suggest that incorporating PSP and CB into the drilling mud formula does not significantly affect density or pH. In contrast, MgO offers additional benefits, including increased density and shale inhibition.

### 3.2. Analysis of PSP, MgO, and CB concentration on formulated mud rheological properties

Evaluated the rheological characteristics of drilling fluid samples using the six-speed Brookfield viscometer method according to API standards. Six individual shear rates were tested during the experiment: 600 rpm, 300 rpm, 200 rpm, 100 rpm, 6 rpm, and 3 rpm, at which shear stress data were acquired once the values became stable. Plastic viscosity (PV, centipoise) was calculated as the difference between shear stress values at 600 rpm and 300 rpm. The yield point (YP) value was calculated by subtracting the plastic viscosity (PV) from the shear stress value at 300 rpm.

Gel 0, or initial gel strength (GS), was measured after the fluid was subjected to high velocity shearing, followed by a 10-second rest period, and gel strength final (Gel 10) was measured after the same procedure, except with a 10-minute rest period. All shear stress values obtained were presented in lbs/100 ft². Identical rheological tests were conducted on each given sample: base mud, nine mud formulations (for S1-S9), and the results are shown in Table 3.

**Table 3:** Brookfield Viscometer Rheological Properties of all 10 drilling mud formulations.

Mud Sample	Unit (SI)	Base Mud	SET A (PSP)			SET B (MgO)			SET C (CB)		
			S1	S2	S3	S4	S5	S6	S7	S8	S9
600 rpm (dial reading)	–	14.8	14.3	25.4	32.5	25.3	32.5	43.2	23.6	32.4	44.3
300 rpm (dial reading)	–	12.7	12.4	18.8	22.4	16.6	22.6	29.5	15.3	20.6	27.6
Plastic Viscosity (PV)	Pa.s	0.0021	0.0019	0.0066	0.0101	0.0087	0.0099	0.0137	0.0083	0.0118	0.0167
Yield Point (YP)	$\frac{kg}{m^{\text{2}}}$	0.5181	0.5132	0.5967	0.6015	0.3968	0.6205	0.7710	0.3420	0.4298	0.5328
Gel Strength, 10 min	$\frac{kg}{m^{\text{2}}}$	3.71	5.47	6.44	7.22	7.08	7.86	9.57	5.66	6.44	10.64
Gel Strength, 10 s	$\frac{kg}{m^{\text{2}}}$	1.76	2.05	2.78	4.05	3.12	4.49	6.98	3.61	4.54	6.73

Rheology of drilling fluids is the most significant factor in determining the haulage potential of cuttings, well-bore stability, and efficient fluid circulation during drilling. The experiment has shown that the addition of PSP, MgO, and CB significantly modifies the plastic viscosity (PV), yield point (YP), and gel strength in the developed drilling fluids. In general, PV, YP, and gel strength also increase with additive concentration, suggesting that the number of particle interactions increases and the drilling fluid structure becomes stronger. PSP, among the additives tested, had a balanced rheological profile. Fibrous and porosity properties of pistachio shell particles increase mechanical interlocking and bridging between particles on the mud matrix. The interactions lead to the creation of a stronger internal structure, improving yield stress and gel strength, which are important for maintaining cuttings suspension in stasis conditions. In addition, PSP is hydrophilic and can therefore partially dissolve in water, increasing both the effective solid content of the suspension and its effective viscosity. Biomass-based additives, such as rice husk ash and lignocellulosic materials, have also been shown to confer rheological advantages to drilling fluids, with fibrous structures observed to enhance viscosity and suspension stability [16]. MgO nanoparticles were found to exhibit significant rheological enhancement, due to their large surface area and strong electrostatic interactions with bentonite particles. The nanoparticles have the potential to occupy interstitial spaces between clay particles and enhance frictional resistance within the suspension, increasing both PV and YP values. As is well known, nanoparticle-based drilling fluids are characterised by a higher degree of rheological stability and hole-cleaning capacity because they enable particles to reinforce the fluid's particle network [24]. However, an overabundance of nanoparticles will lead to overly viscous fluids, and consequently higher pumping rates and energy generation. The additive with the highest viscosity in the test was CB, which had a very small particle size and a strong van der Waals force that favoured particle aggregation. These aggregates create coarse structural systems within the drilling fluid, enhancing resistance to flow and gel strength. Although such behaviour improves suspension stability, it can adversely affect pumpability due to the very thick suspension. The rheological results show that PSP provides a good trade-off between achieving sufficient viscosity for cuttings suspension and maintaining flowability during circulation, making it an efficient, sustainable supplement to water-based drilling fluids.

### 3.3. Effects of PSP, MgO, and CB nanoparticle concentrations on API fluid-loss behaviour

Table 3 displays the fluid-loss characteristics of the formulated drilling mud using the Low Temperature Low Pressure (LTLP) API filter press (OFFITE, U.S.A.) at 1.10 MPa (160 psi) and 30 °C. The three additives were tested at varying concentrations to systematically study their influence on the fluid-loss behavior of the drilling mud formulations. The mud samples were filtered into the filtration cell, using Whatman No. 50 filter paper (or API-standard filter paper). The API fluid loss (filtrate volume) was measured over an incremental time range (4–30 min). The overall volume of the obtained filtrate and the nature of the formed filter cake, in terms of thickness and consistency, were analysed to determine the fluid-loss control performance of the additives. Table 4 reports the experimental results for drilling mud formulations with 2 g additions of PSP, MgO, and CB nanoparticles.

**Table 4:** API filtrate loss at 1.10 MPa (160 psi) and 30 ^°^C for 2 g concentration.

PSP		MgO		CB	
Time (min)	Filtrate Loss (10^−6^ mL)	Time (min)	Filtrate Loss (10^−6^ mL)	Time (min)	Filtrate Loss (10^−6^ mL)
4	8	4	7.5	4	10
8	10.5	8	10	8	14
12	13	12	11.75	12	17
16	15	16	14.5	16	21
20	16.75	20	16.5	20	22
24	18.5	24	17.75	24	24
28	20	28	19	28	25
30	21.25	30	19.75	30	27

**Fig 4:**
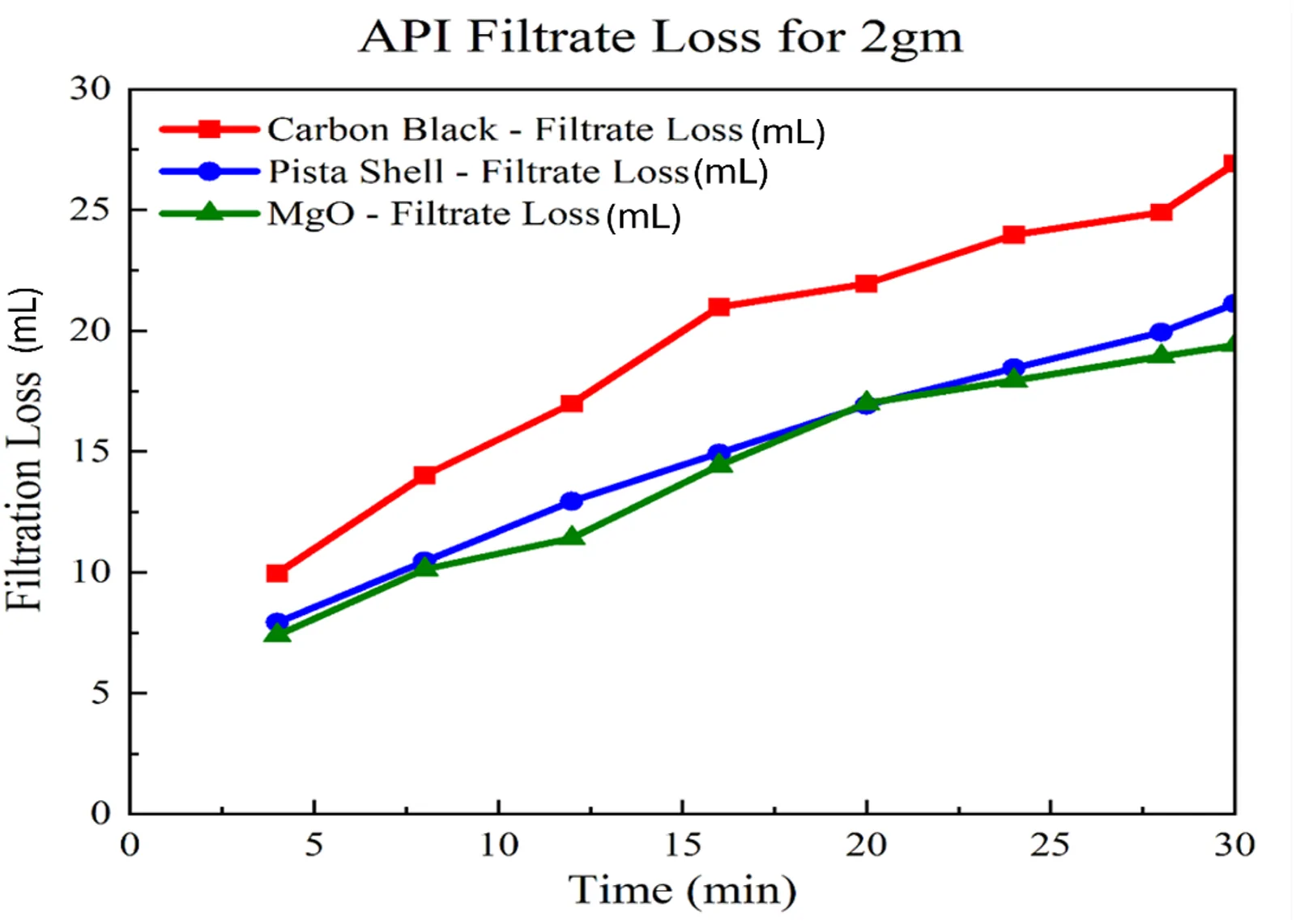
Filtration loss variations for 2 g additions of different additives (PSP, MgO, and CB nanoparticles) to the base mud formulation.

Reducing fluid loss during drilling is a primary requirement for drilling programs. Excessive filtrate entering the formation can lead to formation damage, reduced permeability, and well-bore instability. As shown, the incorporation of the PSP, MgO, and CB particles significantly affects the filtrate loss behaviour of the different drilling mud systems developed, as evidenced by the filtration test conducted with the aid of the API filter press. Fig 4 shows the change in filtrate volume over time at the 2 g additive concentration. It can be seen that the volume of filtrate increases steadily with filtration time in all cases, as shown in the figure, but at different rates depending on the type of additive. MgO-based drilling fluids have the lowest filtrate volume at the end of the 30-minute filtration period (about 19–20 mL), indicating the formation of a tight, low-permeable filter cake. This behaviour is explained by the nano-sized MgO particles, which effectively occupy pore spaces in the filter cake, enhancing particle packing. Previous research also indicates such nanoplastic behaviour; nanoparticles enhance filtration operation by closing micro-pores and fissures in the structure [15]. PSP drilling fluids have somewhat higher filtrate volumes (21 ml), although they still show much better filtration control than CB formulations (26–27 ml). The enhanced performance of the PSP can be attributed to its fibrous, porous structure, which facilitates particle bridging and creates a mechanically stable filter cake.

**Fig 5:**
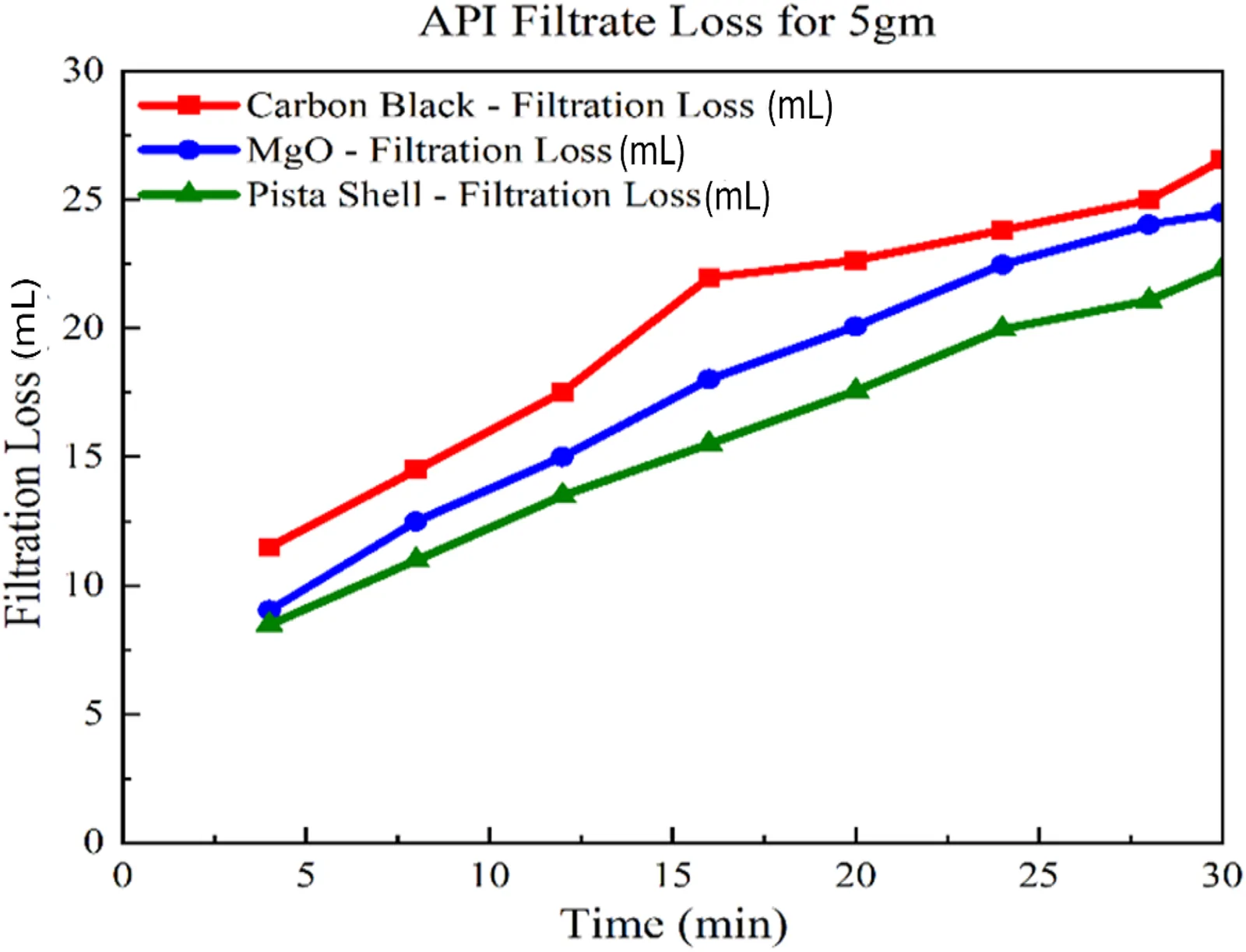
Filtration loss variations for 5 g addition of different additives (PSP, MgO, and CB) to the base mud formulation.

At the higher additive concentration (5 g), the filtration behaviour is shown in Fig 5, and the variations among the additives are more evident. As seen in the figure, PSP exhibits the lowest initial filtrate loss, indicating that it rapidly forms an efficient sealing layer on the filter medium. Such a primitive form of filtration control is particularly significant in drilling processes, as it helps prevent primary fluid intrusion into the formation. MgO systems based on muds exhibit similar performance (approximately 10 mL), whereas CB systems yield relatively higher volumes of filtrate (approximately 11.5 mL). The filtration control observed to be improved with PSP at higher concentrations implies that the fibrous arrangement of the biomass particles improves filter cake formation by forming an interlaced mesh that can trap small particles and increase permeability.

Its hydrophilic nature may also explain PSP's superior performance, as it facilitates water absorption and swelling, thereby enhancing the filter cake's sealing ability. The API filtration and rheological testing were conducted in compliance with API Recommended Practice 13B-1 for Water based drilling fluids. Biomass-based additives used in drilling fluids have also undergone simulated filtration enhancement, with natural fibres and lignocellulosic sources used to enhance the bridging pore geometry and mitigate filtrate intrusion [21]. In contrast, CB particles are poorly filtered because they are hydrophobic and tend to form loosely packed aggregates, resulting in more permeable filter cakes. Generally, the filtration analysis in Figs 4 and 5 supports the significance of additive type and concentration in determining the behaviour of water-based drilling mud systems with respect to fluid loss. The PSP among the discussed additives has the greatest potential to provide optimal filtration control at the product concentration while maintaining environmental friendliness. It is mainly due to physical plugging and bridging effects that accounts for the reduction in filtrate loss. Unlike MgO nanoparticles that reduce permeability by filling up the micro-pores, PSP creates interlaced fibrous network that enhances the compactness of filter cake.

### 3.4. Influence of MgO, CB and PSP particle concentrations on the shear stress, shear strain, and viscosity

Rheological measurements were performed using a compact rheometer in controlled shear rate mode. Drilling mud samples were carefully loaded into the measuring cell to avoid air bubble entrapment. The shear rate was varied from 0.1 to 1000 s ^−1^, and the corresponding shear stress and viscosity were recorded at temperatures of 25°C, 35°C, 45°C, and 50°C. The graphs presented below show the experimental results for the drilling mud formulations with 1 g, 2 g, and 5 g additions of PSP, MgO, and CB nanoparticles with temperature variation (25 ^°^C, 35^o^C, 45^o^C and 50 ^°^C).

**Fig 6:**
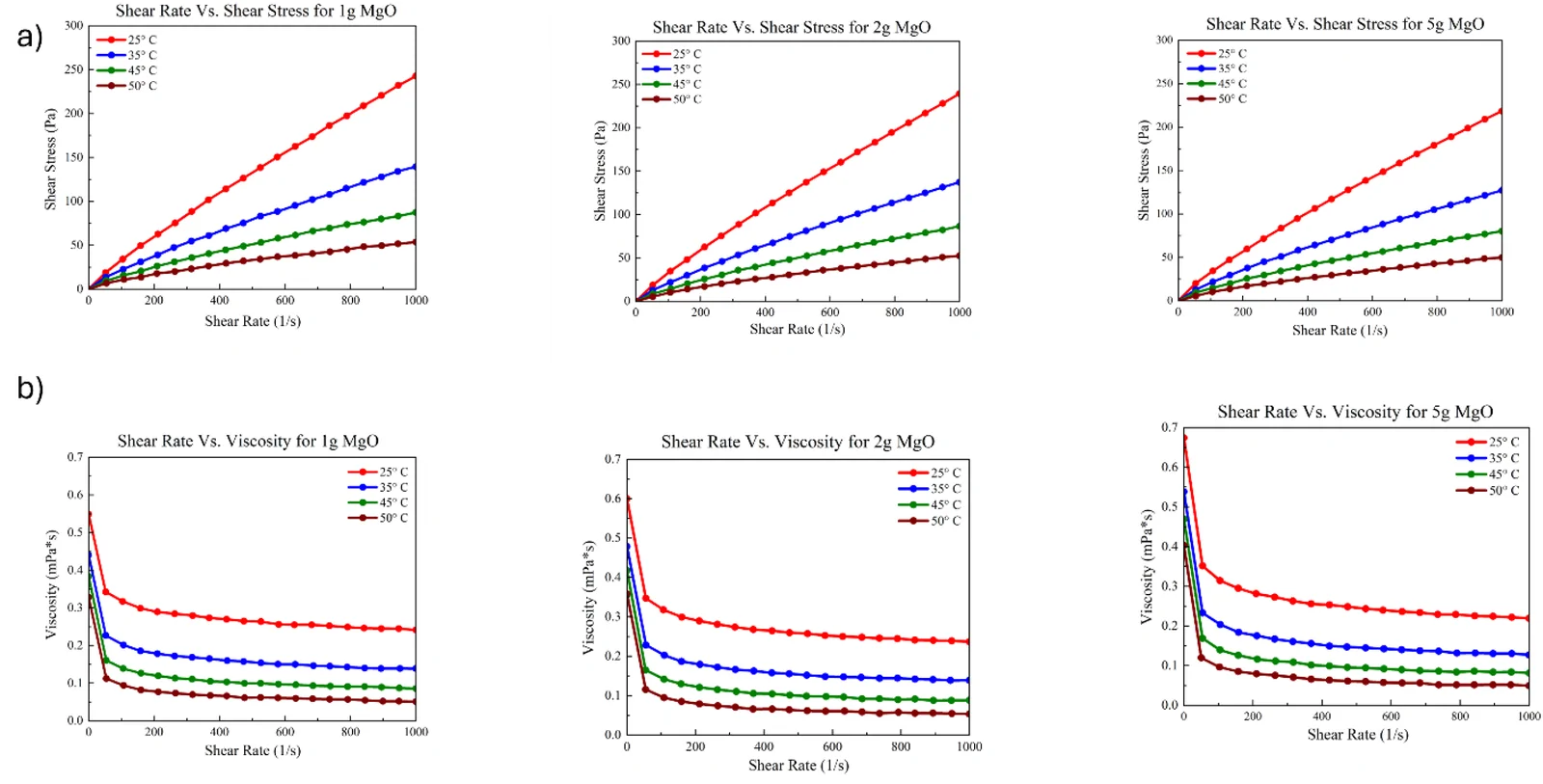
(a) The relationship between shear rate and viscosity of 1 g, 2 g and 5g MgO-based mud at 25 ^°^C, 35^o^C, 45^o^C and 50 ^°^C; (b) the relationship between shear rate and shear stress of 1 g, 2 g and 5g MgO-based mud at 25 ^°^C, 35^o^C, 45^o^C and 50 ^°^C.

**Fig 7:**
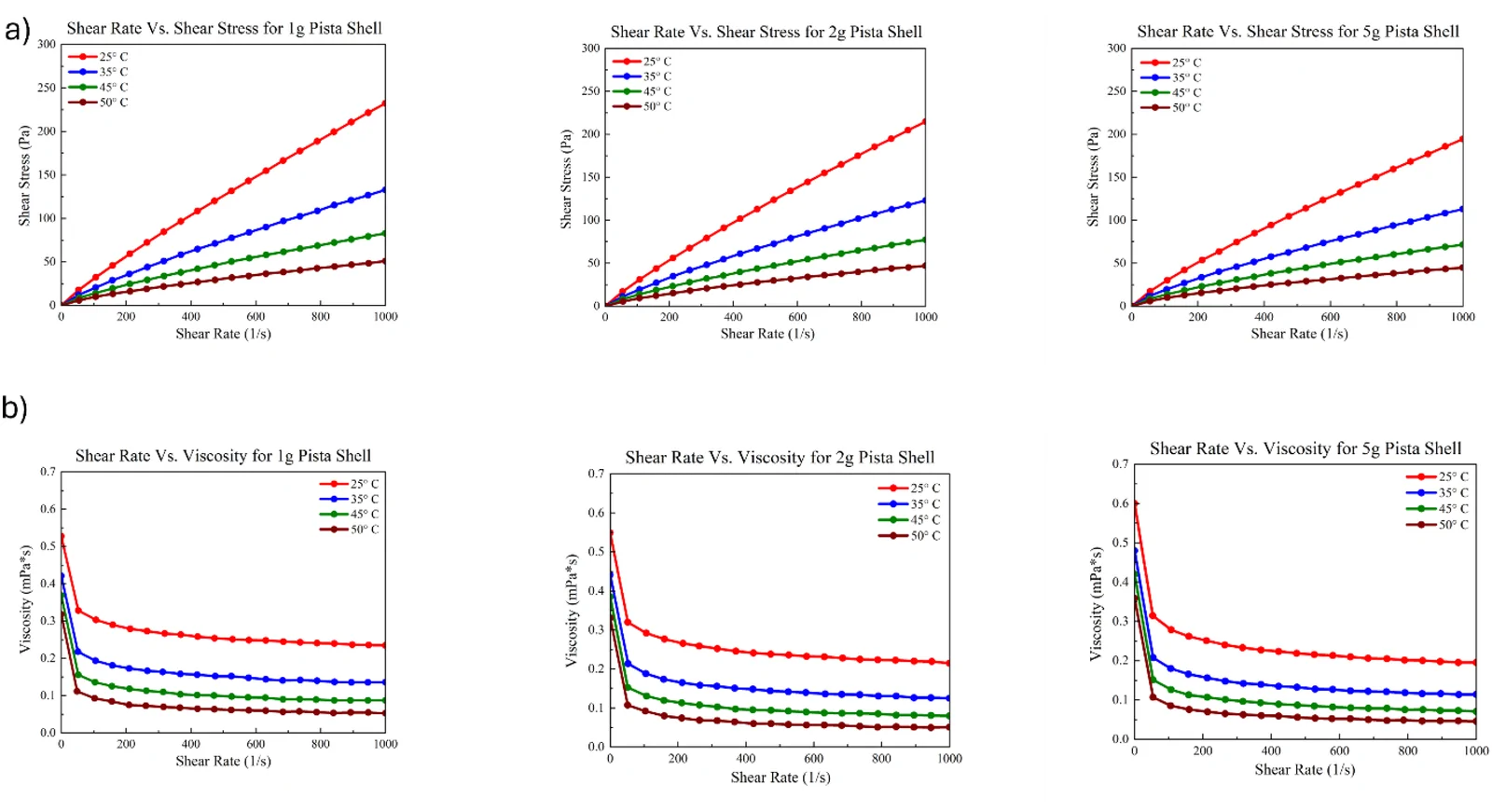
(a) The relationship between shear rate and viscosity of 1 g, 2 g and 5g Pista Shell-based mud at 25 ^°^C, 35^o^C, 45^o^C and 50 ^°^C; (b) the relationship between shear rate and shear stress of 1 g, 2 g and 5g Pista Shell-based mud at 25 ^°^C, 35^o^C, 45^o^C and 50 ^°^C.

**Fig 8:**
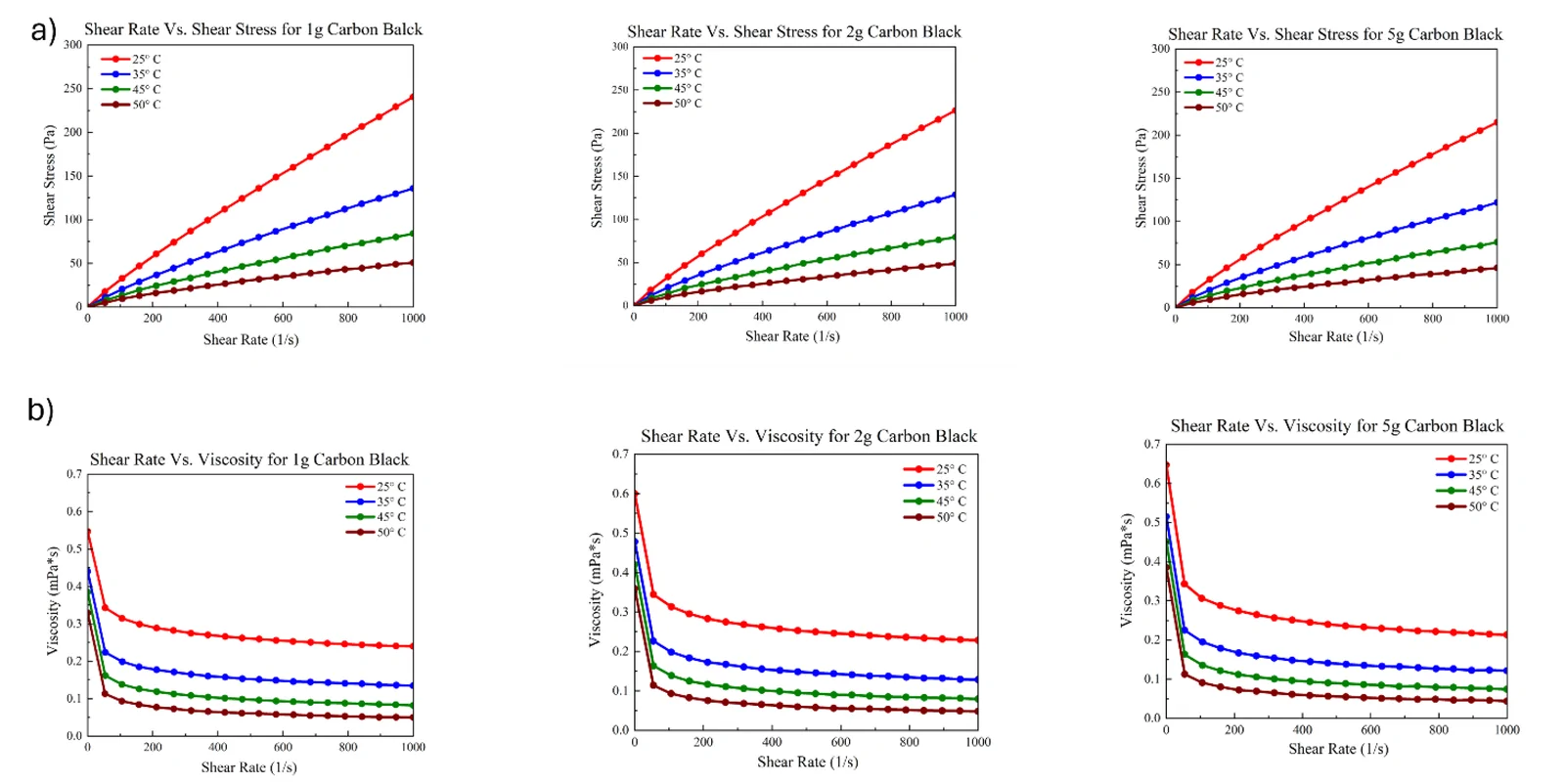
(a) The relationship between shear rate and viscosity of 1 g, 2 g and 5g Carbon Black-based mud at 25 ^°^C, 35^o^C, 45^o^C and 50 ^°^C; (b) the relationship between shear rate and shear stress of 1 g, 2 g and 5g Carbon Black-based mud at 25 ^°^C, 35^o^C, 45^o^C and 50 ^°^C.

The rheological behaviour of the formulated drilling fluids was studied by examining the interactions among shear rate, shear stress, and viscosity at different additive concentrations and temperatures. As shown in Figs 6–8, all the drilling mud systems designed exhibit non-Newtonian shear-thinning behaviour, i.e., viscosity decreases with increasing shear rate. The advantage of this rheological property is that it enables the product to maintain adequate viscosity at low shear rates, suspending cuttings while providing the fluid with minimal resistance to flow during circulation.The rheological behaviour of the MgO nanoparticle-based drilling fluids is shown in Fig 6. The higher the MgO concentration, the higher the viscosity and shear stress. This is probably because MgO nanoparticles have high surface energy and a high surface area, which enhances inter-particle interactions in the drilling fluid. The nanoparticles likely adsorb onto the bentonite platelets, helping to form a robust, deformation-resistant network. This network is not broken at low shear rates, resulting in higher viscosity and shear stress. These particle clusters are disrupted by hydrodynamic forces as the shear rate increases, reducing viscosity. Other nanoparticle-enhanced drilling fluids have been reported to exhibit similar rheological mechanisms, in which nanoparticles support the suspension of clay [14]. Increased MgO levels lead to increased shear stress, an indicator of improved fluid structural integrity and can enhance the suspension's drilling utilisation.

The rheology of PSP-based drilling fluids is shown in Fig 7. The complex particle structure and irregular fibrous morphology of the biomass material increase viscosity when the PSP is included. These particles enhance mechanical particle interlocking and bridging within the drilling fluid matrix, forming a weak yet interlaced structural framework. Compared with spherical nanoparticles, the long fibres of PSP may also entangle to form a network, increasing viscosity and shear resistance at low shear rates. However, as the shear rate further increases, these networks will eventually break apart and orient themselves with the direction of the flow, with the result becoming the shear-thinning response observed in Fig 5. Temperature is also a major factor affecting viscosity. The viscosity decreases with increasing temperature between 25 ^°^C and 50 ^°^C across the various PSP concentrations. This phenomenon is caused by high temperatures, which enhance molecular motion and weaken intermolecular forces in the suspension. The particle networks are larger, and the fluid flows more easily as thermal energy increases. This reduction in the viscosity of biomass-based drilling fluids, additives and colloidal suspensions has been commonly observed to be temperature-dependent [17].

Fig 8 demonstrates the rheological properties of CB-based drilling fluids. Compared with PSP and MgO systems, CB suspensions exhibit higher viscosity over the shear-rate range. This is mainly due to the very small particle size and the high surface area of CB, which facilitates strong van der Waals interactions and particle aggregation. As a result, these aggregates create compacting, percolating structures that build up interior friction and markedly increase resistance to flow. Consequently, CB systems also exhibit higher shear stress values than the other additives. However, it is important to note that such a high tendency toward aggregation may also lead to excessive viscosity, increasing pumping energy requirements during drilling. Furthermore, Fig 6 shows that rising temperature decreases viscosity by disrupting these aggregated structures thermally and reducing their interactions with one another.

The comparison of Figs 6–8 demonstrates the influence of particle shape and surface features on drilling fluid flow. MgO nanoparticles increase thickness by adsorbing onto clay and acquiring negative charges, whereas CB increases thickness by agglomerating and forming networks at the fluid surface. PSP enhances fluid flow by binding to and entangling with other particles. These structural variations account for the variations in thickness and flow resistance. It is worth noting that fluids in PSP are neither too thick nor too thin; however, when stirred, they become much easier to pump, which helps remove material from the drill hole. The rheological and filtration enhancement of PSP is consistent with previous investigations of rice husk ash, walnut shell powder, and various cellulose-based additives, as the fibrous and porous structure of biomass enhances the bridging and integrity of the filter cake. Compared with these materials, PSP exhibited comparable filtration-reducing capabilities and good drilling-fluid rheological properties, suggesting strong potential as an environmentally friendly drilling-fluid additive.

## 4. Conclusions

This study systematically compared the performance of pistachio shell powder (PSP), a sustainable agricultural by‑product, with magnesium oxide (MgO) nanoparticles and carbon black (CB) as additives in water‑based drilling muds. The results demonstrate that PSP significantly enhances rheological properties, increasing the yield point to 0.771 kg/m² and the gel strength to 19.6 lb/100 ft² while maintaining a stable density (9.82–9.88 ppg) and pH (8.0–8.2), indicating excellent compatibility with the base mud. In filtration tests, PSP achieved an API filtrate volume of 22 mL, superior to CB (26–27 mL) and comparable to MgO, by forming a thin, low‑permeability filter cake, attributed to its fibrous morphology and hydrophilic nature. All mud systems exhibited non‑Newtonian shear‑thinning behaviour, with PSP providing an optimal balance between sufficient viscosity for cuttings suspension and flowability during circulation. Notably, PSP, derived from agricultural waste, offers a cost‑effective, environmentally friendly alternative to conventional additives, performing on par with MgO nanoparticles while avoiding the high energy requirements associated with CB’s excessive thickening. Considering these results, PSP can be used as a viable biodegradable fluid-loss control agent for water-based drilling fluids operation, particularly in environmentally sensitive areas for which environmentally friendly drilling fluids are needed. This work is aligned with the Sustainable Drilling Technologies (SDGT) for SDGs 7, 9, 12, 13: valorization of agricultural waste materials into environmentally responsible drilling fluid additives. The present work gives valuable information about the influence of additive type (PSP, MgO and CB), but only within the scope of laboratory-scale LTLP conditions and the use of additives up to 5 g. These were not the areas of investigation of this work but should be explored in future studies: HTP drilling environment, long-term stability of suspensions, formation damage, permeability effects, and microstructural characterization of filter cakes (SEM/TEM) and investigation of settling tendency of PSP particles. The future plans for the study should include HTHP performance studies, field scale validation, synergistic combinations of PSP with nanoparticles, reservoir permeability evaluation and optimization of concentration of additives for advanced drilling applications.
